# Nuclear Factor I-C Regulates Stemness Genes and Proliferation of Stem Cells in Various Mineralized Tissue through Epithelial-Mesenchymal Interactions in Dental Epithelial Stem Cells

**DOI:** 10.1155/2022/1092184

**Published:** 2022-09-27

**Authors:** Dong-Seol Lee, Yeo Joon Song, Hye Ri Gug, Ji-Hyun Lee, Hyun Sook Bae, Joo-Cheol Park

**Affiliations:** ^1^Laboratory for the Study of Regenerative Dental Medicine Department of Oral Histology-Developmental Biology, School of Dentistry and Dental Research Institute, Seoul National University, 1 Gwanakro, Gwanak-Gu, Seoul 08826, Republic of Korea; ^2^Regenerative Dental Medicine R and D Center, Hysensbio Co., Ltd., Seoul, Republic of Korea; ^3^Department of Oral Hygiene, Namseoul University, Cheonan, Republic of Korea

## Abstract

Tooth development includes numerous cell divisions and cell-cell interactions generating the stem cell niche. After an indefinite number of divisions, pluripotent cells differentiate into various types of cells. Nuclear factor I (NFI) transcription factors are known as crucial regulators in various organ development and stem cell biology. Among its members, nuclear factor I-C (NFI-C) has been reported to play an essential role in odontogenesis. *Nfic* knockout mice show malformation in all mineralized tissues, but it remains unclear which stage of development Nfic is involved in. We previously reported that *Nfic* induces the differentiation of ameloblast, odontoblast, and osteoblast. However, the question remains whether *Nfic* participates in the late stage of development, perpetuating the proliferation of stem cells. This study aimed to elucidate the underlying mechanism of NFI-C function in stem cells capable of forming hard tissues. Here, we demonstrate that *Nfic* regulates Sox2 and cell proliferation in diverse mineralized tissue stem cells such as dental epithelial stem cells (DESCs), dental pulp stem cells, and bone marrow stem cells, but not in fibroblasts. It was also involved in the expression of pluripotency genes Lin28 and NANOG. Especially in DESCs, *Nfic* regulates the proliferation of epithelial cells via epithelial-mesenchymal interactions, which are the Fgf8-Nfic-Sox2 pathway in epithelium and Nfic-Fgf10 in the mesenchyme. Moreover, *Nfic* slightly increased reprogramming efficiency in induced pluripotent stem cells of mineralized tissues, but not in soft tissues. In conclusion, these results suggest that *Nfic* is a crucial factor for maintaining the stem cell niche of mineralized tissues and provides a possibility for *Nfic* as an additional factor in improving reprogramming efficiency.

## 1. Introduction

The lifelong-growing feature of rodent incisors comes from the active stem cell population. Stem cell niche is maintained through reciprocal interaction between neural crest-originated ectomesenchyme and ectodermal epithelial stem cells [1, 2]. The specific epithelial structure called the cervical loop is in the very apex of the incisor. The cervical loop consists of stellate reticulum (SR) surrounded by inner enamel epithelium (IEE) and outer enamel epithelium (OEE). In the labial side of the rodent incisor, dental epithelial stem cells (DESCs) migrate from SR to IEE and generate the rapid-dividing transit-amplifying (TA) cells. After dividing a finite number of times, TA cells differentiate into pre-ameloblasts that move to the incisor's distal tip during maturation and eventually become ameloblasts to secrete enamel matrix proteins. The stem cell niche of DESCs is maintained through epithelial-mesenchymal interactions [3–5].

The nuclear factor I (NFI) is a family of proteins that bind to specific DNA sequences to regulate gene expression, which acts as transcription factors essential for developing various organ systems [6]. Among the four members of the NFI family (NFI-A, NFI-B, NFI-C, and NFI-X), NFI-C is known to play an essential role in odontogenesis. *Nfic*^−/−^ mice show defects in several mineralized tissues such as short, defective incisors, absence of roots in molars, and low bone density [7]. Such defects in enamel, dentin, and bone imply the significance of *Nfic* in mineralized tissue development and maintenance. Furthermore, the mRNA expression levels of tooth-specific genes, including amelogenin, ameloblastin, and dentin sialophosphoprotein (DSPP), decreased significantly in *Nfic*^−/−^ mice mandible [8].

The development begins with numerous cell divisions, generating the stem cell niche. After an indefinite number of divisions, pluripotent cells differentiate into tissue-specific types. We previously reported that *Nfic* induces the proliferation and differentiation of ameloblast, odontoblast, and osteoblast [7, 9, 10]. *Nfic* was also reported to promote the proliferation of apical papilla-derived stem cells (SCAP) [11]. However, the question remains whether *Nfic* is involved in the former stage, such as maintaining the proliferation of stem cells. Our previous experiments demonstrated that *Nfic* is involved in the self-renewal of stem cells in cartilage [12]. However, there had been no report on other mineralized tissues, such as enamel, dentin, and bone, which warrants further research to understand the effect of Nfic on self-renewal in these stem cells.

Sox2 belongs to a family of SRY-related homology box transcription factors, which are essential during development and cellular differentiation [13]. Sox2 has been identified as an epithelial stem cell marker in mouse incisors that play a crucial role in maintaining stem cell pluripotency [14–17]. While Sox2 deficient embryos lack a pluripotent ICM and fail early in development, deletion of Sox2 in embryonic stem cells (ESCs) results in their improper differentiation into trophectoderm-like cells [18]. Recent studies have shown that conditional deletion of Sox2 using Pitx2-cre caused incisor arrest at embryonic day (E) 16.5 and regression at E18.5, as well as incisor growth defects after its conditional deletion in the embryonic incisor epithelium using *ShhGFP − cre/+* [19, 20]. Sox2 is also required to maintain the self-renewal and multilineage in the osteoblasts progenitor cells [21, 22]. Liu et al. (2015) have shown that Sox2 overexpression in hDPSCs improved cell proliferation, migration, and adhesion [23]. Furthermore, forced expression of Sox2, in tandem with Oct4, Klf4, and c-Myc, endows somatic cells with pluripotency, giving rise to induced pluripotent stem cells (iPSCs) [24]. Although such findings indicate the importance of Sox2 in establishing and maintaining pluripotent stem cells, its relationship with intercellular proteins and their gene regulatory network during stem cell proliferation and differentiation are not clearly understood.

Fibroblast growth factors (Fgf) signaling has a crucial role in inducing the proliferation and differentiation of multiple cell types during embryonic stages. It has also regulated mouse tooth development [25, 26]. Several members of the Fgf family are expressed in early developing tooth germ. From tooth initiation to cusp formation, they play an essential role in distinct stages of odontogenesis [27]. Fgf8 is expressed in the dental epithelium before tooth initiation and persists until the early bud stage, restricting tooth forming sites by inducing the expression Pax9, Pitx1, and Pix2 [28]. Fgf8 is also speculated to be responsible for the induction of Fgf3 expression in dental mesenchyme. Both Fgf3 and Fgf10 expressions are detected in the dental papilla mesenchyme adjacent to the epithelial of the cervical loop, which acts as mesenchymal signals regulating epithelial morphogenesis [29]. Studies have shown defective enamel in Fgf3^−/−^ mice and very thin or absent enamel in Fgf3^−/−^; Fgf10^+/-^ mice alongside data examining incisors developing *in vitro* have suggested that Fgf10 regulates epithelial stem cell survival [30–32]. However, a comprehensive mechanism underlying the FGFs that regulate incisor renewal in adulthood is still poorly understood.

This study aims to elucidate the role of *Nfic* in stem cell proliferation in enamel, dentin, and bone. Especially in dental epithelial cells, the relationship between *Nfic*, Sox2, and associated signaling molecules was studied from the perspective of epithelial-mesenchymal interactions. Moreover, the capacity of *Nfic* was evaluated as an additional reprogramming factor that escalates the efficiency of iPSCs.

## 2. Materials and Methods

### 2.1. Reagents and Antibodies

Recombinant human FGF-8 (423-F8) and Shh (1845-SH) were purchased from R&D Systems (R&D Systems, Minneapolis, MN). Antisera against NFI-C were produced as described previously [9]. Rabbit cyclin D1 (#2922) was purchased from Cell Signaling Technology (Cell Signaling Technology, Danvers, MA). Antibodies against PCNA (SC-7907), p21 (sc-6246), Cytokeratin 14 (sc-53253), and Sox2 (sc-365964) were purchased from Santa Cruz Biotechnology (Santa Cruz Biotechnology, Dallas, TX).

### 2.2. Animals

All mice experiments were performed according to the Dental Research Institute guidelines and the Institutional Animal Care and Use Committees of Seoul National University (SNU-181127-13-2). *Nfic*^−/−^ mice that were generated by removal of the second exon from *Nfic* gene were kindly provided by Dr. Richard M. Gronostajski [6], and homozygous *Nfic*^−/−^ mice were obtained by crossing male and female heterozygous *Nfic*^+/-^ mice. As *Nfic*^−/−^ mice have brittle teeth, a ground standard rodent chow was provided to all animals three times a week beginning 3 days before weaning and continued for up to 6 weeks [7].

### 2.3. Micro-CT Analysis, Histology, and Immunohistochemistry (IHC)

The mandible and tongue from 6-weeks wild type (WT) and *Nfic*^−/−^ mice were removed and fixed in 4% paraformaldehyde at 4 °C overnight. The mandibles were analyzed by micro-CT with a SkyScan scanner and the associated software (Skyscan 1172, Kontich, Belgium). Decalcified mandibles and tongues were sectioned and subjected to hematoxylin-eosin (H&E) staining and IHC. IHC was performed as previously described [7].

### 2.4. Cell Culture and Transfection

Apical bud cells (ABCs) were isolated and cultured from the cervical loop of lower incisors of WT and *Nfic*^−/−^ mice. The lower incisors were separated from WT and *Nfic*^−/−^ mice at postnatal day 7. ABCs were enzymatically isolated from cervical loop tissues and cultured in a keratinocyte serum-free medium (K-SFM, Invitrogen, Carlsbad, CA) until reaching confluence. Primary pulp cells were isolated and cultured from lower incisors of WT and *Nfic*^−/−^ mice as previously described [9]. Briefly, after the incisors were dissected out, they were cracked longitudinally using a 27-gauge needle on a 1-ml syringe. The pulp tissues were removed gently with forceps, cut into several pieces, and placed on 60-mm culture dishes (Nunc). The explants were weighed down with a sterile cover glass and cultured in Dulbecco's modified Eagle's medium (Invitrogen) supplemented with 100 IU/ml penicillin, 100 IU/ml streptomycin (Invitrogen), and 10% fetal bovine serum (Invitrogen).

The ameloblast-lineage cell line (ALCs), provided by Dr. T. Sugiyama (Akita University School of Medicine, Akita, Japan), was cultured in MEM containing 5% FBS and supplemented with 10 ng/ml recombinant human epidermal growth factor (EGF; 20 ng/ml, R&D Systems, Minneapolis, MN). The expression plasmids for *Nfic* and si*Nfic* were prepared as described previously [7]. The expression plasmid for Sox2 was purchased from OriGene (MR204615) (OriGene, Rockville, MD), and each of those expression plasmids (2 *μ*g) was transiently transfected into ALCs using the Lipofectamine Plus™ reagent (Invitrogen, Carlsbad, CA) according to the manufacturer's instructions.

Tongue epithelial cells were isolated from 6-weeks WT and *Nfic*^−/−^ tongues. Dissected tongues were placed in Dispase II (1.6 mg/ml) (Invitrogen, Carlsbad, CA) at 37 °C for 30 min and separated into tongue epithelium and mesenchyme. The tongue epithelial tissues were incubated in 1% collagenase/Dispase II solution (Invitrogen, Carlsbad, CA) at 37 °C for 1 hr, and the tissues were dissociated by gently pipetting up and down. The cells were cultured in a keratinocyte serum-free medium (K-SFM, Invitrogen, Carlsbad, CA). Tail fibroblasts and dermal fibroblasts were isolated from 6-weeks WT and *Nfic*^−/−^ tails and skins. Dissected tissues were placed in 1% collagenase/Dispase II solution (Invitrogen, Carlsbad, CA) at 37 °C for 1 hr, and the tissues were dissociated by gently pipetting up and down. The cells were cultured in DMEM supplemented with 100 IU/ml penicillin, 100 *μ*g/ml streptomycin, and 10% FBS.

Human dental pulp stem cells (hDPSCs) were established as previously described [33]. Impacted human third molars from patients between the ages of 18 and 22 were provided by the Seoul National University Dental Hospital. The experimental protocol was approved by the Institutional Review Board (IRB No: S-D20140007). Informed consent was obtained from every patient. Isolation of whole pulp cells was performed as previously described, and cells were cultured in minimum essential media *α* (MEM-*α*; Gibco BRL) for use in *in vitro* and *ex vivo* experiments.

Human bone marrow stem cells (hBMSCs) were purchased from Cambrex (Cambrex Corporation, East Rutherford, NJ) and cultured in low DMEM supplemented with 100 IU/ml penicillin, 100 *μ*g/ml streptomycin, and 10% FBS.

### 2.5. MTT Assays

The proliferation of the cells was evaluated using MTT assays. Cells were seeded and cultured on 96-well plates at a density of 3 × 10^3^ cells/well. After washing with PBS, 50 *μ*l of MTT (5 mg/ml) was added to each well and incubated for 4 hrs at 37 °C. After removing the MTT solution, the converted dye was dissolved in DMSO and measured by reading the absorbance at a wavelength of 540 nm with a microplate reader (Multiskan EX, Thermo Fisher Scientific Inc., Waltham, MA). Triplicate samples were analyzed from two independent experiments.

### 2.6. Luciferase Assays

A 0.766-kb genomic fragment was amplified from mouse genomic DNA to obtain the Sox2 promoter (−528 to +238). Mouse genomic DNA (1 *μ*l) was subjected to PCR using the following cycling conditions: 94 °C, 1 min; 60 °C, 1 min; and 72 °C, 1 min for 35 cycles. The forward and reverse primers were as follows: Sox2 Fw 5′- GCT AAG CTT GTG CTG GCG ACA AGG TTG GAA GAG GGG C-3′, Sox2 RV 5′- GCG CTC GAG CAA TTG GGA TGA AAA AAC AGG C-3′, Sox2 Mut_Fw 5′- AGC CGG CGC TCG CTG CAG CTG TAT CGG AAA CCC ATT TAT TC-3′, and Sox2 Mut_Rv 5′- GAA TAA ATG GGT TTC CGA TAC AGC TGC AGC GAG CGC CG-3′. The amplified fragment was subcloned into the PCR®2.1 T vector (Invitrogen, Carlsbad, CA) and ligated into the XhoI and Hind III sites of the pGL3 luciferase (LUC) basic expression vector (Promega, Madison, WI). Luciferase assays were performed as described previously [9]. Briefly, for each transfection, 0.4 *μ*g of the luciferase reporter plasmid was used along with 0.4 *μ*g of the expression vector, as indicated. The vectors used were pGL3basic and pGL3 Sox2 promoter (−528 to +238). Cells were also incubated for 48 hrs with FGF-8 (10 ng/ml). After incubation for 48 hrs, cells were assessed for luciferase activity using the luciferase reporter gene assay system (Roche Applied Science, Indianapolis, IN) according to the manufacturer's instructions. Measurements were performed using a luminometer (FLUOStar OPTIMA, BMC Laboratory, Offenburg, Germany).

### 2.7. ChIP Assays

ChIP assays were performed as previously described [7]. Briefly, ALCs were transiently transfected with NFI-C expression plasmid for 48 hrs. The samples were sonicated, followed by chromatin immunoprecipitation with anti-NFIC (30 *μ*l) and anti-IgG (10 *μ*l) (Santa Cruz Biotechnology, Dallas, TX) antibodies. The final DNA pellets were recovered and analyzed by PCR using specific primers. PCR primers were synthesized as listed in Table 1. The following PCR conditions were used: 94 °C for 30 s; 60 °C for 30 s; and 72 °C for 30 s for a total of 35 cycles. The PCR products were electrophoresed in a 2% agarose gel, stained with ethidium bromide, and visualized under ultraviolet light.

### 2.8. Reverse Transcription-Polymerase Chain Reaction (RT-PCR) and Real-Time PCR Analyses

Total RNA (2 *μ*g) was reverse transcribed using 0.5 *μ*g of Oligo (dT) [9] and 1 *μ*l (50 IU) of Superscript III enzyme (Invitrogen, Carlsbad, CA) in a 20 *μ*l reaction mixture at 50 °C for 1 hr. The resulting mixture was amplified by PCR. For real-time PCR, specific primers for *Nfic*, Sox2, FGF-10, *Lin28, Nanog*, and *Gapdh* were synthesized as listed in Table 2. Real-time PCR was performed on an ABI PRISM 7500 sequence detection system using the SYBR GREEN PCR Master Mix (Applied Biosystems, Foster City, CA) according to the manufacturer's instructions. The PCR conditions were 94 °C for 1 min, followed by 95 °C for 15 s and 60 °C for 34 s for 40 cycles. All reactions were run in triplicate and normalized to the housekeeping gene, *Gapdh*. Relative differences in PCR results were calculated using the comparative cycle threshold (C_T_) method.

### 2.9. Western Blot Analyses

Western blot analyses were performed as previously described [9]. Briefly, proteins (30 *μ*g) were separated by 10% sodium dodecyl sulfate-polyacrylamide gel electrophoresis, transferred onto a nitrocellulose membrane (Schleicher & Schuell BioScience, Inc., Keene, NH), and labeled with specific antibodies. Labeled protein bands were detected using an enhanced chemiluminescence system (GE Healthcare, Chicago, IL).

### 2.10. Generation of hiPSCs

Generation of hiPSCs was performed as previously described [34]. Briefly, pMX-retroviral vectors coding for human Oct4, Sox2, Klf4, and c-Myc (Addgene, Watertown, MA), Nfic, and packaging vectors pCMV-VSVG were co-transfected into GP2-293 cells using calcium-phosphate mammalian transfection kit (Takara Bio USA, Inc., San Jose, CA). 48 and 72 hrs after transfection, the virus-containing supernatants were collected, filtered (0.45-mm filter, MilliporeSigma, Burlington, MA), and concentrated through ultracentrifugation (Beckman Coulter, Brea, CA). For the hiPSC generation, hDPSCs were seeded at a density of 1 × 10^5^ cell per well in a six-well plate. 24 hours later, the cells were infected with the virus and maintained with the respective primary cell culture medium in the presence of 8 mg/ml of polybrene (Sigma-Aldrich, St. Louis, Missouri, MO). Five days post-transduction, the cells were reseeded and further cultured in a modified hESC medium after adding 1 mM nicotinamide. The medium was changed every other day until hESC-like colonies emerged. Reprogramming efficiency was quantified with the number of ALP-positive colonies.

### 2.11. Statistical Analysis

All quantitative data are presented as the mean ± S.D. Statistical differences were analyzed using the Student's *t* tests (^∗^*p* < 0.05).

## 3. Results

### 3.1. *Nfic* Level is Reduced as the DESCs Differentiate into Ameloblasts

**Figure 1:**
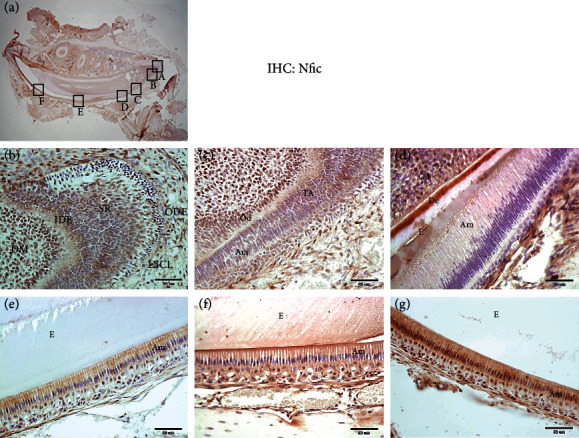
*Nfic* expression level decreases as DESC differentiate into ameloblasts. (a) Immunohistochemistry (IHC) analysis of the sagittal section of a WT mandibular incisor is examined to observe the expression pattern of *Nfic* in the labial cervical loop. The histological analysis of the labial cervical loop shows the organized structure with DESCs differentiating into ameloblasts towards the cervical loop of the incisor. (B)–(F) are higher magnifications of boxed (A), from right to left, respectively. (A)–(F) 6 weeks. Scale bars = (B)–(E) = 50 *μ*m. (b–g) Illustrated diagram of the *Nfic* expression pattern and differentiation level in the cervical loop of the mandibular incisor. Abbreviations: Am: ameloblast; D: dentin; DM: dental mesenchyme; E: enamel; IEE: inner enamel epithelium; IHC: immunohistochemistry; Od: odontoblast; OEE: outer enamel epithelium; SR: stellate reticulum; TA: transit-amplifying cells; WT: wild type.

*Nfic*-deficient mice show malformation in bone and teeth. We again confirmed that *Nfic*^−/−^ mice had osteoporotic bone (Supp. Figures 1 (a) and 1(b)) and underdeveloped cervical loop (Supp. Figures 1(c)–1(h)) at 6 weeks using *μ*CT and H&E staining. The IEE, SR, and OEE layers in the cervical loop were highly disrupted, barely discernible in the *Nfic*^−/−^ mice. The DESCs in peripheral SR, particularly those close to the vicinity of basal epithelial cells, become IEE. Interestingly, the expression of *Nfic* was the strongest in the labial cervical loop, which is the reservoir of epithelial stem cells (Figure 1(a: A,B)). The *Nfic* level gradually decreased as the differentiation proceeded through SR, TA cells to secretory ameloblasts, and then its level gradually increased in the transition stage to the maturation stage ameloblast (Figures 1(a: B–F) and 1(b)–1(g)). Given that differentiation requires stemness genes to be turned off, these results suggest that *Nfic* may correlate with the stemness of dental epithelial cells.

### 3.2. *Nfic* Regulates Sox2 Expression in Dental Epithelium

**Figure 2:**
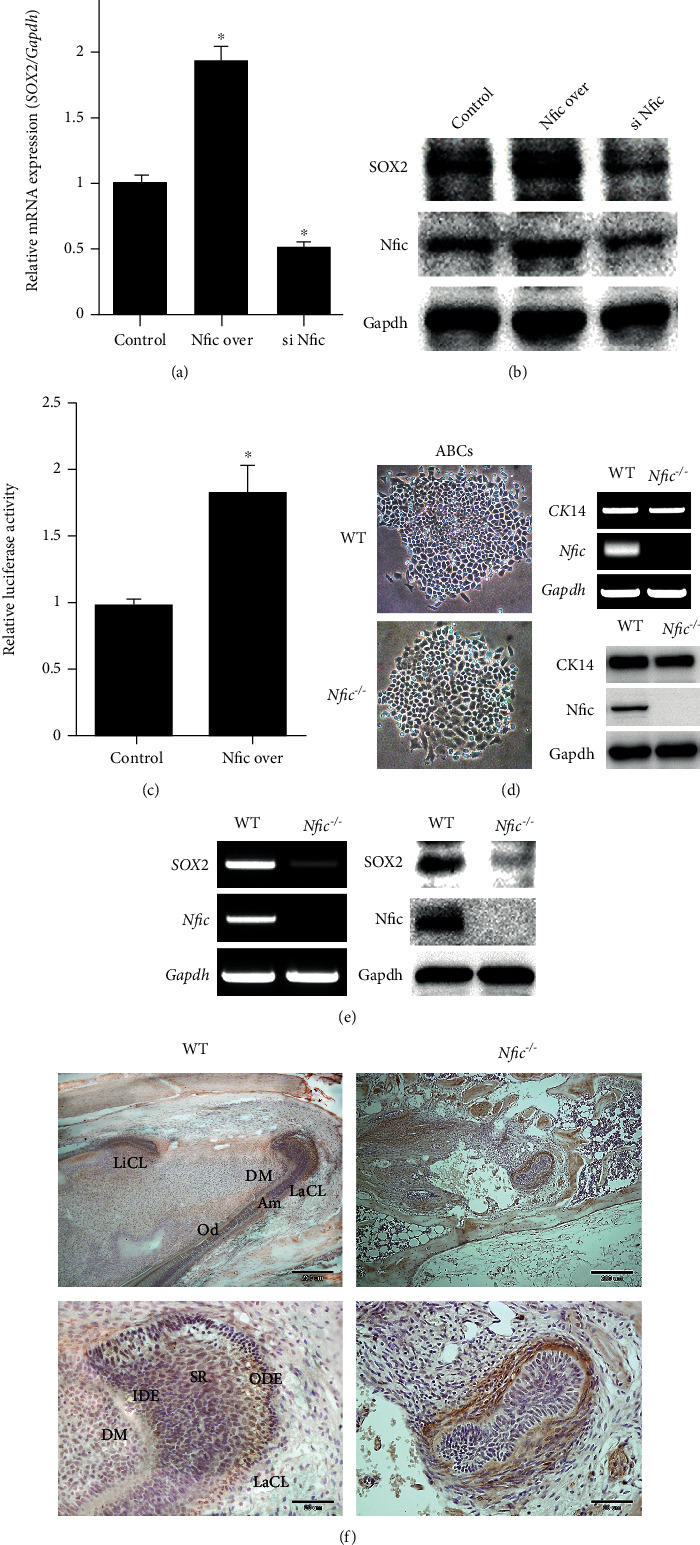
*Nfic* regulates Sox2 expression in the dental epithelium. (a) Expression of Sox2 mRNA and (b) protein levels were analyzed in the transfected ALCs. *n* = 5, ^∗^*p* < .05. (c) Sox2 promoter activity was analyzed in the *Nfic*-overexpressing ALCs in comparison to the control. *n* = 5, ^∗^*p* < .05. (d) Sox2 gene and (e) protein expression levels in ABC from the cervical loop of WT and *Nfic* KO mice were analyzed. (f) The histological structure of WT and *Nfic* KO cervical loop and their Sox2 expression pattern were examined through immunohistochemistry (IHC) staining. (b) and (d) are higher magnifications of boxed (a) and (c), respectively. Postnatal 7 days. Scale bars = (a) and (c) = 200 *μ*m; (b) and (d) = 50 *μ*m. Data are presented as the mean ± SD. Abbreviations: ALC: ameloblast lineage cell; CL: cervical loop; DM: dental mesenchyme; IEE: inner enamel epithelium; IHC: immunohistochemistry; KO: knockout; OEE: outer enamel epithelium; SR: stellate reticulum; WT: wild type.

Sox2 has been suggested as a stem cell marker of the dental epithelium [4, 17, 35, 36]. Ameloblast lineage cells (ALCs) were transfected with *Sox2*, *Nfic*-overexpressing, and si*Nfic plasmids* to determine whether Sox2 level depended on *Nfic*. Sox2 mRNA and protein levels depended on *Nfic* (Figures 2(a) and 2(b)). In addition, the promoter activity of Sox2 was significantly higher in *Nfic*-overexpressing ALCs (Figure 2(c)).

ABCs are the pluripotent cells harvested from the labial cervical loop of the rodent incisor. To determine whether ABC was isolated from the cervical loop, we checked the cell morphology of the isolated ABCs by light microscopy. In addition, the expression of CK14, an epithelial cell marker gene, was analyzed by RT-PCR and western blot. Isolated ABCs from WT and *Nfic*^−/−^ mice showed epithelial morphology, and expression of ck14 was also detected (Figure 2(d)). These primary pluripotent cells from *Nfic* KO mice showed downregulated Sox2 gene and protein expressions (Figure 2(e)). In the cervical loop of *Nfic* KO mice form postnatal 6-weeks, the organization of dental mesenchyme, SR, IEE, and OEE was disrupted with decreased Sox2 expression, mainly in the SR and IEE. However, the expression of Sox2 in the incisor of 6-week-old wild-type mice was strongly expressed OEE on the cervical loop and weakly detected in the dental mesenchyme and dental follicle (Figure 2(f: a–d)). *Nfic* mRNA level in ALCs where Sox2 was overexpressed remained unchanged (data not shown). These results support the hierarchical relationship between *Nfic* and Sox2.

### 3.3. Loss of *Nfic* Decreases the Proliferation of Dental Epithelial Cells

**Figure 3:**
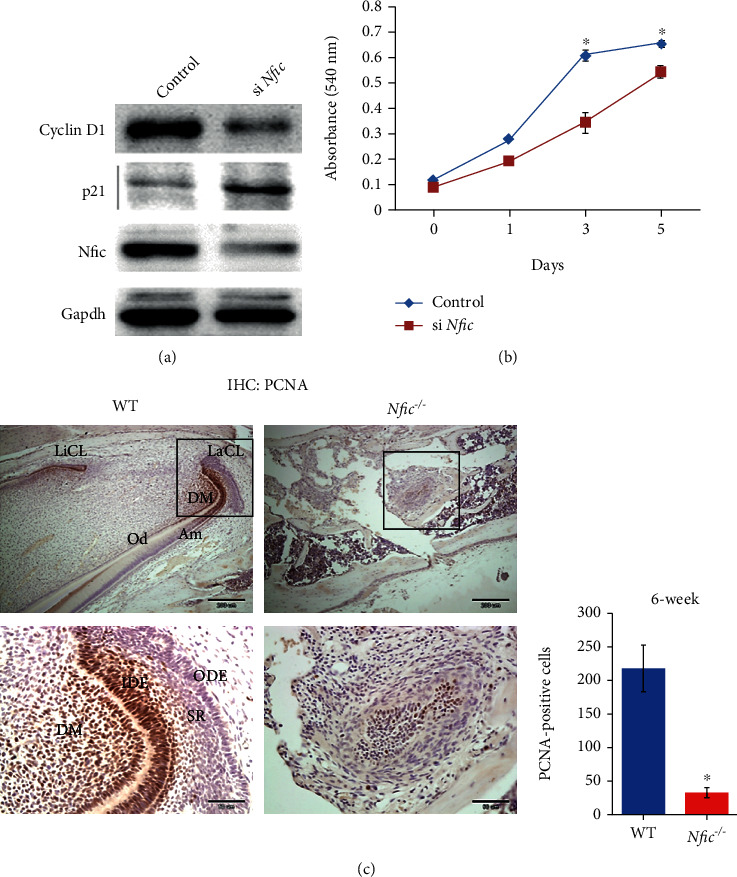
Loss of *Nfic* decreases the proliferation of dental epithelial cells. (a) Western blot analyses to evaluate the expression levels of cyclin D1 and p21 in control and *Nfic*-silenced cells. (b) The proliferation rate of control and *Nfic*-silenced cells from day 0 to 5. *n* = 5, ^∗^*p* < .05. (c) Immunohistochemistry (IHC) staining analysis of PCNA-positive cells in the cervical loop of the (a, b) control and (c, d) *Nfic*-silenced mice. (e) The number of PCNA-positive cells in the cervical loop of these mice after 6 weeks. *n* = 5, ^∗^*p* < .05. (b) and (d) are higher magnifications of (a) and (c), respectively. Scale bars = (a) and (c) = 200 *μ*m; (b) and (d) = 50 *μ*m. Data are presented as the mean ± SD. Abbreviations: DM: dental mesenchyme; IEE: inner enamel epithelium; OEE: outer enamel epithelium; SR: stellate reticulum; TA: transit-amplifying cells; WT: wild type.

Self-renewal is another key feature of stemness that sustains a level of cell population with constant proliferation. Conditional inactivation of *Sox2* in oral and dental epithelium has shown that Sox2 is crucial in the proliferation and maintenance of DESCs [20]. Since *Nfic* regulates Sox2 expression, *Nfic* was expected to be involved in the proliferation and maintenance of DESCs. As predicted, cell cycle marker cyclin D1 diminished and p21 incremented when *Nfic* was silenced, indicating that cell proliferation had shifted to a quiescent state in the absence of *Nfic* (Figure 3(a)). The proliferation rate of *Nfic*-silenced ALCs was significantly lower than the control group, especially between days 1 and 3 (Figure 3(b)). The number of PCNA-positive cells in the cervical loop of *Nfic* KO mice was nearly one-fifth of the WT (Figure 3(c)). Altogether, these results support that *Nfic* modulates the maintenance of DESCs and the proliferation of dental epithelium.

### 3.4. Not Shh, but FGF8 is the Upstream Signaling Molecule for the Nfic-Sox2 Pathway in Dental Epithelial Cells

**Figure 4:**
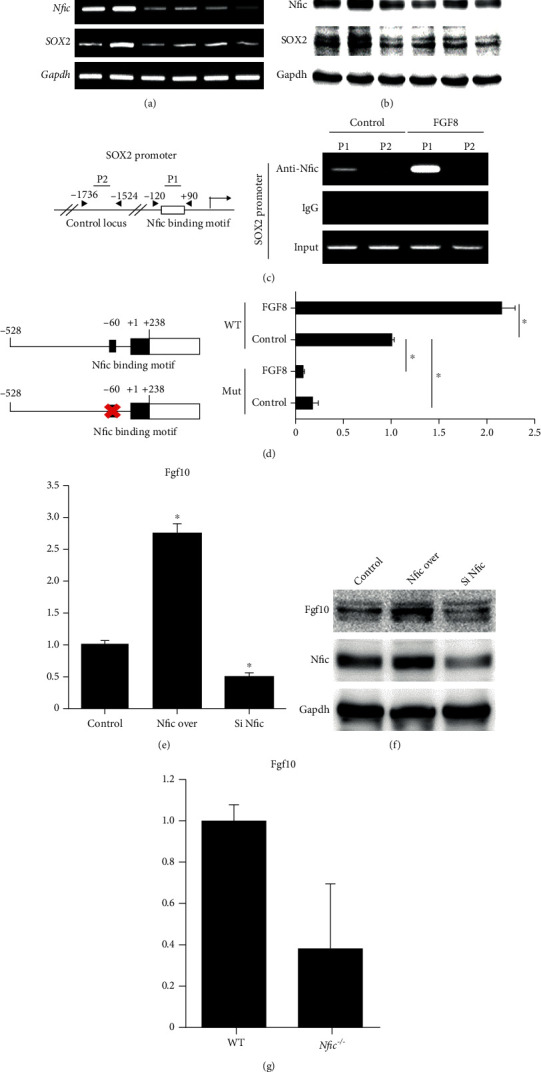
FGF8 is the upstream signaling molecule for *Nfic*-Sox2 in dental epithelial cells, and *Nfic* induces FGF10 in the dental mesenchymal stem cells to modulate the proliferation of DESC through epithelial-mesenchymal interactions. (a) Sox2 protein and (b) mRNA expression levels were analyzed when treated with FGF8 and Shh in the control and *Nfic*-silenced dental epithelial cells. (c) Schematic representation of Sox2 promoters and (d) the deletion of the *Nfic* binding motif. The effect of FGF8 on the Sox2 promoter activity in the absence of the *Nfic* binding motif is shown. *n* = 5, ^∗^*p* < .05. (e) Fgf10 mRNA and (f) protein expression levels were examined when *Nfic* was overexpressed or silenced. (g) FGF10 expression levels were analyzed in the primary cultured dental pulp cells from *Nfic* KO mice. *n* = 5, ^∗^*p* < .05. (h) Schematic diagram of hypothesized intercellular interactions involved in stem cell homeostasis in dental epithelial cells. Data are presented as the mean ± SD. Abbreviations: FGF: fibroblast growth factor; LUC: luciferase; Over: overexpression; Shh: sonic hedgehog; WT: wild type.

FGF8 and Shh are the representative upstream molecules of Sox2 in dental epithelial cells [17, 37, 38]. When treated with FGF8, both Sox2 mRNA and protein levels were elevated in ALCs (Figures 4(a) and 4(b)). However, when *Nfic* was silenced, there was no change in the Sox2 expression even with the FGF8 treatment. By contrast, Shh treatment did not change the Sox2 expression (Figures 4(a) and 4(b)). Moreover, adding FGF8 increased Nfic binding to the cognate DNA binding site in the Sox2 promoter (Figure 4(c)). When the *Nfic* binding motif in the Sox2 promoter was deleted, FGF8 could not upregulate the activity of the Sox2 promoter (Figure 4(d)). These molecular data confirm FGF8 to be located upstream of the Nfic-Sox2 signaling pathway.

### 3.5. *Nfic* Induces FGF10 in Dental Mesenchymal Stem Cells, which Modulates the Proliferation of DESCs by Epithelial-Mesenchymal Interactions

Maintaining the DESC niche in the cervical loop requires complex reciprocal interactions between epithelial and mesenchymal cells [4, 29]. In the cervical loop of the rodent incisor, Fgf10 from dental mesenchyme regulates the proliferation of the stem cell niche [29, 32]. The mRNA and protein levels of Fgf10 were associated with *Nfic* expression in mouse dental pulp cells (MDPCs) (Figures 4(e) and 4(f)). Also, primary cultured dental pulp cells from *Nfic* KO mice showed lower Fgf10 expression levels than WT (Figure 4(g)).

These results support the hypothesis that *Nfic* modulates the epithelial-mesenchymal interactions known to maintain the DESCs. Within the dental epithelium, it mediates *Fgf8–Nfic–Sox2* signaling pathway, and in the dental mesenchymal stem cells, *Nfic* induces Fgf10 which is known to regulate the proliferation of dental epithelial stem cells (Figure 4(h)).

### 3.6. *Nfic* Also Regulates Sox2 Expression and Cell Proliferation in Stem Cells from Other Mineralized Tissue, DPSCs and BMSCs

As previously mentioned, *Nfic* KO mice show defects in every mineralized tissue. Enamel, dentin, bone, and cartilage are the hard tissues in vertebrates, each formed by ameloblasts, odontoblasts, osteoblasts, and chondroblasts, respectively. Our previous experiments showed that *Nfic* regulates cell proliferation and differentiation in chondroblasts (data not shown). Therefore, we speculated whether *Nfic* plays a crucial role in the proliferation of stem cells from dentin and bone, specifically DPSCs and BMSCs.

**Figure 5:**
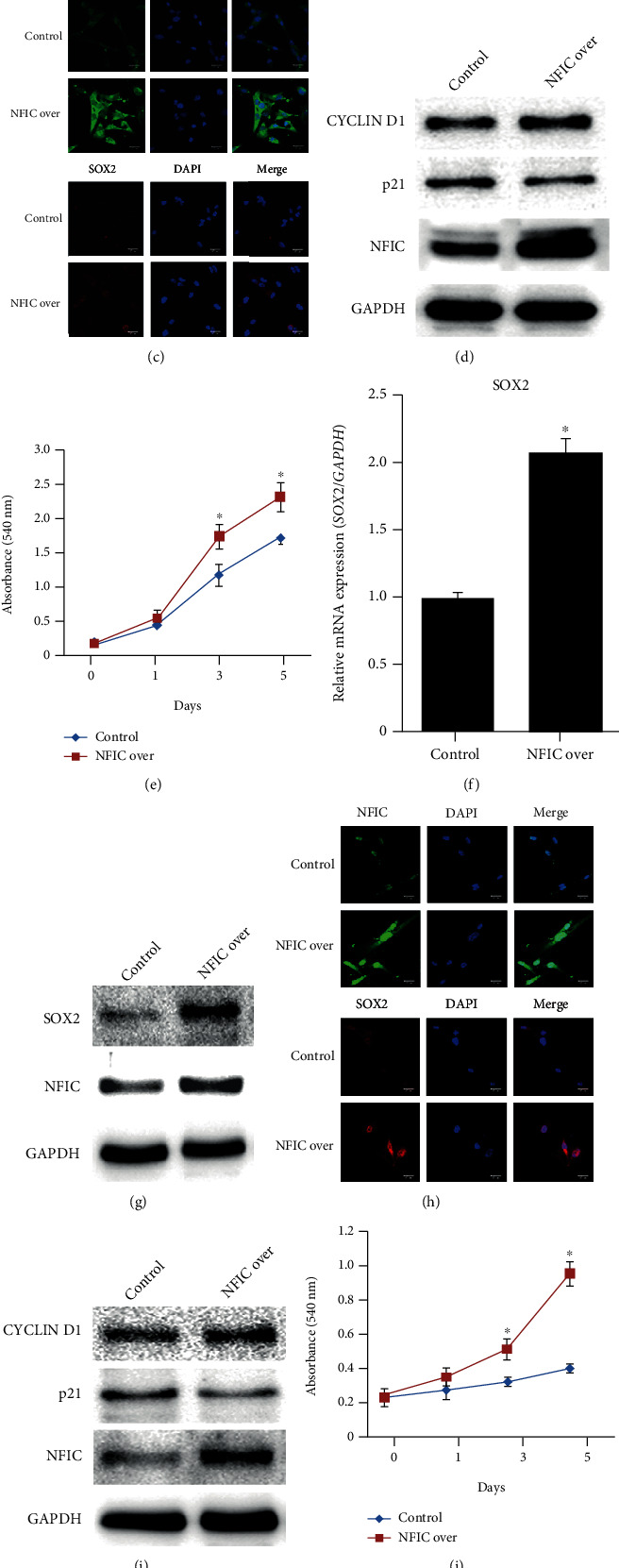
*Nfic* regulates Sox2 expression in DPSC (a–e) and BMSC (f–j). (a) Sox2 mRNA and (b) protein expression levels were analyzed in control and *Nfic*-overexpressing DPSC. *n* = 5, ^∗^*p* < .05. (c) Sox2 expression *in vitro* examined in cultured DPSC under fluorescence microscopy. (d) Western blot analyses to evaluate the expression levels of cyclin D1 and p21 in DPSC. (e) Cell population of DPSC from day 0 to 5 with increased *Nfic* expression. *n* = 5, ^∗^*p* < .05. (f) Sox2 mRNA and (g) protein expression levels were analyzed in control and *Nfic*-overexpressing BMSC. *n* = 5, ^∗^*p* < .05. (h) Sox2 expression *in vitro* was examined in cultured BMSC under fluorescence microscopy. (i) Western blot analyses to evaluate the expression levels of cyclin D1 and p21 in BMSC. (j) Cell population of BMSC from days 0 to 5 with increased *Nfic* expression. *n* = 5, ^∗^*p* < .05. (k) Immunohistochemistry (IHC) analysis of Sox2 expression in mouse femur *in vivo*. 6 weeks. Scale bars = 20 *μ*m. Data are presented as the mean ± SD. Abbreviations: BMSC: bone marrow stem cell; DPSC: dental pulp stem cell; IHC: immunohistochemistry; Over: overexpression; WT: wild type.

Overexpression of *Nfic* increased *Sox2* mRNA and protein expression in DPSCs and BMSCs (Figures 5(a), 5(b), 5(f), and 5(g)). The intensity of Sox2 staining in *Nfic*-overexpressing DPSCs and BMSCs was stronger compared to the control group (Figures 5(c) and 5(h)). The cell cycle markers cyclin D1 and p21 also shifted to a proliferative state (Figures 5(d) and 5(i)). DPSCs and BMSCs showed a significantly higher cell population with increased *Nfic* expression (Figures 5(e) and 5(j)). Moreover, *Nfic* ablation decreased Sox2 expression in mouse femur *in vivo* (Figure 5(k)). In summary, our data corroborate that *Nfic* regulates Sox2 expression and stem cell proliferation in various mineralized tissues.

### 3.7. *Nfic* Modulates Pluripotency Gene Expression in Mesenchymal Stem Cells of Mineralized Tissue

**Figure 6:**
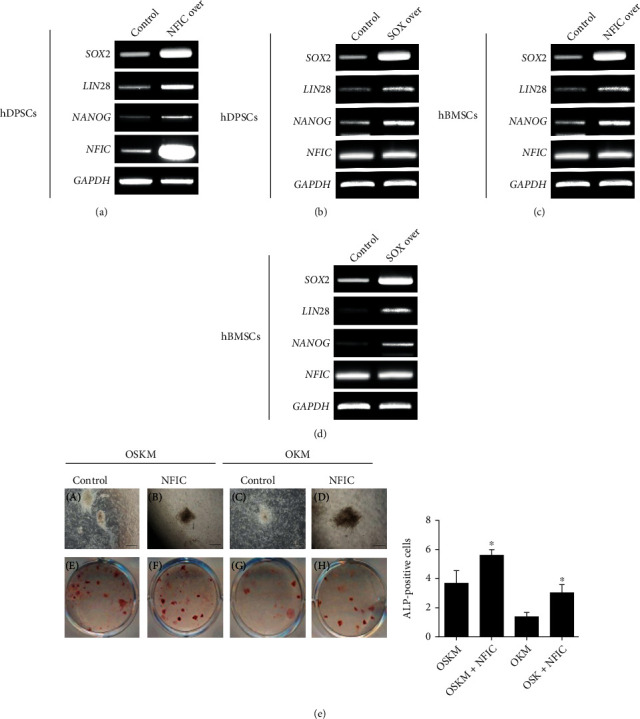
*Nfic* modulates pluripotency gene expression in mesenchymal stem cells of mineralized tissues. (a) Lin28 and Nanog gene expression levels were analyzed in control, *Nfic*-overexpressing, and (b) Sox2-overexpressing DPSC. (c) Lin28 and Nanog gene expression levels were analyzed in control, *Nfic*-overexpressing, and (d) Sox2-overexpressing BMSC. (e) (A–D) The number of ALP-positive colonies of iPSC cultured in OSKM medium compared to the addition of *Nfic*. (E–H) The number of ALP-positive colonies of iPSC cultured in OKM medium compared to the addition of *Nfic*. Scale bars = (A), (C), (E), (G) = 200 *μ*m. (f) Quantitative analysis of the number of ALP-positive colonies in various conditioned media. Data are presented as the mean ± SD. Abbreviations: ALP: alkaline phosphatase; BMSC: bone marrow stem cell; Ctrl: control; DPSC: dental pulp stem cell; iPSC: induced pluripotent stem cell; OSKM, Oct4, Sox2, Klf4, c-Myc; OKM, Oct4, Klf4, c-Myc.

A recent study has reported that NANOG and LIN28 improved OSKM-mediated reprogramming efficiency and reduced latency period [39]. Both Nanog and Lin28 were higher in *Nfic*- and *Sox2*-overexpressing group compared to the control group (Figures 6(a)–6(d)).

Since *Nfic* modulates one of the reprogramming factors for iPSCs, we speculated whether *Nfic* could enhance reprogramming efficiency. The addition of *Nfic* to OSKM showed a slight increment in the number of ALP-positive colonies (Figures 6(e) and 6(f)). Moreover, the addition of *Nfic* to OKM resulted in a colony number with no significant difference compared to OSKM without the addition of *Nfic* (Figure 6(f)). Collectively, *Nfic*-treatment to OKM showed comparably improved efficiency, which provides evidence for *Nfic* to be a supporting factor for iPSCs.

### 3.8. *Nfic* Affects Sox2 Expression Only in Mineralized Tissues, Not Soft Tissues

**Figure 7:**
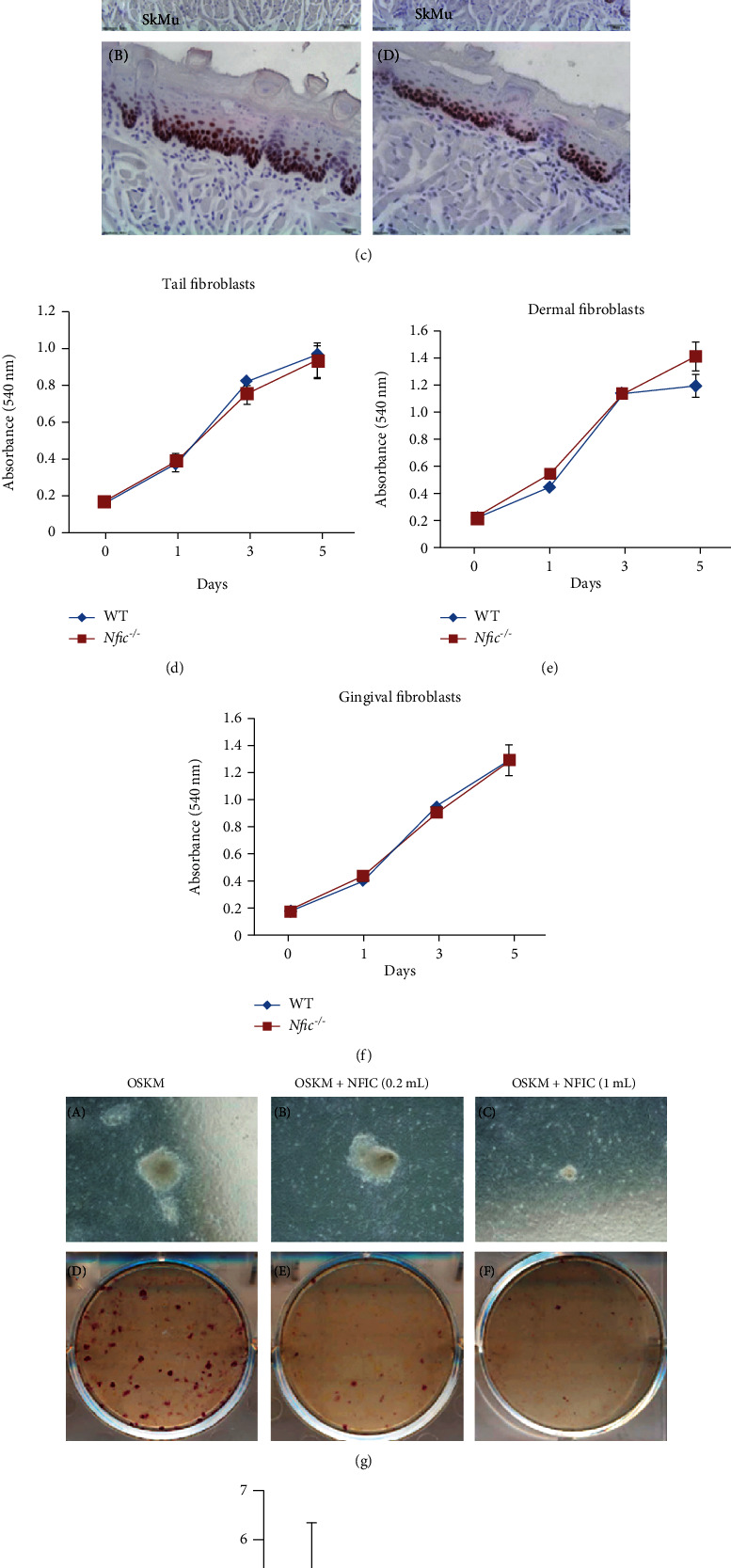
*Nfic* does not affect Sox2 expression in soft tissues. (a) Sox2 mRNA and (b) protein expression levels were analyzed in primary cultured fibroblasts harvested from the tongues of *Nfic*^−/−^ mice. (c) Immunohistochemistry (IHC) analysis of Sox2 expression in the tongues. (B) and (D) are higher magnifications of boxed (A) and (C). 6 weeks. Scale bars = (A), (C) = 50 *μ*m; (B), (D) = 20 *μ*m. (d) Proliferation rate of tail, (e) gingival, and (f) dermal fibroblasts from *Nfic*^−/−^ mice from day 0 to 5 compared to the WT. *n* = 5, ^∗^*p* < .05. (g) The number of ALP-positive colonies of iPSC from fibroblasts cultured in OSKM medium compared to the addition of *Nfic* in a dose-dependent manner. (A), (C), and (E) are higher magnifications of (B), (D), and (F), respectively. (h) Quantitative analysis of the number of ALP-positive colonies in various conditioned media. Data are presented as the mean ± SD. Abbreviations: ALP: alkaline phosphatase; Epi: epithelium; IHC: immunohistochemistry; iPSC: induced pluripotent stem cell; LaPr: lamina propria; OSKM, Oct4, Sox2, Klf4, c-Myc; OKM, Oct4, Klf4, c-Myc; SkMu, skeletal muscle; SqEp: squamous epithelium; WT: wild type.

Reprogramming iPSCs was established with the fibroblasts [24]. Fibroblasts were harvested from the tongues of *Nfic* KO mice (Supplementary Figure 2). Sox2 mRNA and protein expression levels in primary cultured fibroblasts were indistinguishable from that of WT (Figures 7(a) and 7(b)). Moreover, *Nfic* disruption did not affect Sox2 expression in the squamous epithelium where stem cells reside (Figure 7(c)). Moreover, fibroblasts from various soft tissues of *Nfic* KO mice showed an undistinguishable proliferation rate compared to WT (Figures 7(d)–7(f)). Surprisingly, *Nfic* suppressed the number of ALP-positive colonies in a dose-dependent manner (Figures 7(g) and 7(h)). In summary, *Nfic* is involved in Sox2 expression and cell proliferation in the mineralized tissues but not in the soft tissues.

## 4. Discussion

As development ends, the significance of stem cells is shifted onto regeneration. Self-renewal is the constant division of stem cells perpetuating the regenerative capacity throughout life. This distinct feature of stem cells permits them to reconstitute the stem cell pools after acute injuries and attenuated self-renewal can lead to degenerative diseases [40–42]. Therefore, identification of the factors involved in self-renewal is invaluable.

In the present study, we demonstrated that *Nfic* regulates Sox2 in the rest of mineralized tissue forming cells such as ameloblasts, odontoblasts, and osteoblasts. We have found that *Nfic* is involved in cell proliferation, stem cell niche maintenance, and cell fate determination through regulating Sox2 not only in DESCs but also in DPSCs and BMSCs. Furthermore, *Nfic* slightly accelerated the iPSC efficiency in DESCs but not in fibroblasts. Our results have again verified the *Nfic* as the critical factor for the proliferation of stem cells in mineralized tissues.

Our *in vivo* data have shown the ablation of *Nfic* reduced the Sox2 expression in mouse ABCs. Previously, genetic inducible fate mapping demonstrated Sox2 positive cells as adult stem cells and the source of every dental epithelial cell lineage in the mouse incisor. However, since the mouse cervical loop has various cells, there could be other *Nfic*-positive cells other than the Sox2-positive ones. It is known that the expression of Sox2 in the mouse incisor is mainly expressed in the outer dental epithelium (OEE) and stratum intermedium (SI). However, Li Zhang et al. reported that sox2 mRNA expression was strongly expressed in OEE and SI but weakly expressed in dental mesenchyme and dental follicle [43, 44]. In addition, Peng et al. reported that Sox2 was expressed in the dental papilla and dental follicle in a time-dependent manner [45, 46]. We confirmed that level of Sox2 protein in P14-old wild-type mice was strongly expressed in OEE and SI but not in the dental mesenchyme and dental follicle (data not shown). However, Sox2 in the incisor of 6-week-old wild-type mice was strongly expressed in OEE and SI but was weakly detected in the dental mesenchyme and dental follicle. Therefore, our results suggest that similar to previous reports, Sox2 may be expressed differently in a time-dependent manner. Therefore, further specification of cell types is necessary to confirm their relationship.

**Figure 8:**
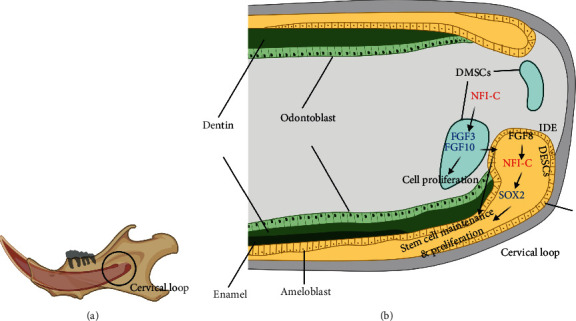
Schematic diagram demonstrating the signaling pathway in the context of ectomesenchymal interaction for maintenance and proliferation of DESCs in mouse incisor cervical loop. *Nfic* is involved the cell proliferation and maintenance of DESCs in two different pathways. Within the dental epithelium, it mediates FGF8-NFIC-SOX2 signaling pathway. In the dental mesenchymal stem cell, Nfic induces FGF10 which controls the proliferation of dental epithelial stem cells.

Earlier investigations have reported *Nfic* as essential for odontogenic and osteogenic cell proliferation, but the exact signaling was unknown [9, 47]. Here, we demonstrated that *Nfic* controls the self-renewal of DESCs through epithelial-mesenchymal interactions, specifically via signaling pathways Fgf8-Nfic-Sox2 in DESCs and Nfic-Fgf10 in dental mesenchymal stem cells (Figure 8). The tooth initiation marker Fgf8 has been reported to control Sox2 with specific miRNAs to fine-tune dental epithelium [17]. In the cervical loop of the rodent incisor, *Fgf3* and *Fgf10* from dental mesenchyme are associated with proliferation of dental epithelium and homeostasis of stem cell niche. The *Fgf10* KO mice show a severely reduced cervical loop that is similar to that of *Nfic* KO mice. In addition, FGF8, not FGF10, is crucial for *Sox2* expression in the incisor cervical loop [17], and *Fgf8* and its receptor *Fgfr1c* were co-localized with *Sox2* in the cervical loop [38]. Remarkably, a single transcription factor *Nfic* modulates both epithelial and mesenchymal signals.

Besides its critical role in epithelial-mesenchymal interactions, *Nfic* is also involved in expressing pluripotency genes Lin28 and Nanog. Wang et al. (2019) showed the synergistic effect of NANOG and LIN28 to improve and reduce the latency of reprogramming [39]. This explains the improvement in reprogramming efficiency in the OSKM+Nfic group (Figures 6(e) and 6(f)). The results strengthen the notion that *Nfic* is an integrating factor of epithelial and mesenchymal regulation on the maintenance of the stem cell niche in the cervical loop of the dental epithelium.

Shu et al. (2013) have suggested a “seesaw model” wherein the balanced equilibrium between the pluripotency factors and/or lineage specifiers facilitates the induction of pluripotency, which emphasizes the significance of interactions between various factors [48]. In addition, Oct4 was revealed to interact with the downstream markers such as Sox2, Nanog, Klf4, and c-Myc to initiate reprogramming [49]. It has been shown that Oct4 and Nanog maintained the self-renewal ability and inhibited differentiation in mesenchymal stem cells [50]. As far as we know, no report has demonstrated the correlation between *Nfic* and Oct4. Since *Nfic* controls the expression of Sox2, and Oct4 is another crucial factor for the pluripotency or the lineage specification, it would be worth investigating the correlation between *Nfic* and Oct4.

*Nfic* accelerated the proliferation ALP positive colonies even without the presence of Sox2. The reprogramming efficiency of OKM + Nfic was similar compared to that of OSKM. As *Nfic* regulates Sox2 expression, the absence of Sox2 being rescued by adding *Nfic* is consistent with our understanding. As of recently, almost all of the pluripotency genes, including the Yamanaka factors (OSKM), Nanog, and Lin28, can be replaced by other molecules [51]. Therefore, the possibility of *Nfic* being an additional factor in augmenting reprogramming efficiency should still be considered. As *Nfic* regulates skeletal cell proliferation and is involved in inducing multipotency, we speculate that *Nfic* may be utilized in making pluripotent stem cells from primary skeletal cells. However, further evaluations on the quality of *Nfic*-induced iPSCs, such as their multipotency and chimera formation, is necessary to support the hypothesis better.

In contrast to its major involvement in cell proliferation and differentiation in mineralized tissues, *Nfic* did not affect either the Sox2 expression or cell proliferation in soft tissues, including tail, dermal, and gingival fibroblasts. It is likely that *Nfic* interacts differently in soft tissues and does not regulate Sox2 at all or in the same manner. Furthermore, we initially predicted that *Nfic* introduction would have no effect on the colony formation cultured in OSKM factors as *Nfic* did not affect the Sox2 expression levels (Figures 7(a) and 7(b)). Surprisingly, the number of ALP-positive colonies cultured in OSKM factors decreased with the addition of *Nfic*, and the decline was more evident with a higher dosage of *Nfic* (Figures 7(g) and 7(h)). One explanation is the Krüppel-like factor 4 (Klf4), which is regulated by *Nfic*, and known to promote dentinogenesis through Dmp1 and DSPP expression. Overexpression of *Nfic* upregulated Klf4 in odontoblast and promoted mineralized nodule formation [52]. A recent study has reported that KLF4 is an antifibrotic factor in fibroblasts and fibrotic genes were highly expressed in KLF4-KO dermal fibroblasts [53]. These two pieces of evidence provide a possible justification of the phenomenon as higher dosage of *Nfic* upregulated Klf4 in the ALP positive colonies and Klf4 with an antifibrotic property decreased the number of colonies in a dose-dependent manner in various fibroblasts. However, the exact relationship between *Nfic* and Klf4 in soft tissues and other confounding factors contributing to decreasing differentiation in fibroblasts warrants further investigation.

## 5. Conclusion

We have demonstrated that *Nfic* plays a crucial role in maintaining the stem cell population in DESCs, DPSCs, and BMSCs by regulating Sox2 expression. Contrarily, *Nfic* showed no effect on either the Sox2 expression or cell proliferation in various fibroblasts. *Nfic* participated in preserving the epithelial stem cell population via epithelial-mesenchymal interactions in the cervical loop of the rodent incisor. We also verified the possibility of *Nfic* as an additional factor to augment the reprogramming efficiency of iPSCs from DPSCs.

## Figures and Tables

**Table 1:** Nucleotide sequences of ChIP assay PCR primers.

Gene		Primer (5′-3′)
Sox2 promoter P1	Forward ( −122)	CCTTTCATGCAAAACCCTCT
	Reverse (+90)	CCTAGTCTTAAAGAGGCAGC

Sox2 promoter P2	Forward (−1736)	TGGGGAAGAGGCGTTGGTGG
	Reverse (−1524)	AATAAAGAGCTTAAACCATC

**Table 2:** Nucleotide sequences of real-time PCR primers.

Gene		Primer (5′-3′)
mNfic	Forward	GACCTGTACCTGGCCTACTTTG
	Reverse	CACACCTGACGTGACAAAGCTC

mSox2	Forward	TAGAGCTAGACTCCGGGCGATGA
	Reverse	TTGCCTTAAACAAGACCACGAAA

mFgf10	Forward	CTGGAAAGCACTTGGGTCAT
	Reverse	GGAGACAGAATGCACAAGCA

mCK14	Forward	TACTTCAAGACCATTGAGGAC
	Reverse	TCATGCGCAGGTTCAACTCT

mGapdh	Forward	AGGTCGGTGTGAACGGATTTG
	Forward	TGTAGACCATGTAGTTGAGGTCA

hSOX2	Forward	GACTTCACATGTCCCAGCAC
	Forward	GGGTTTTCTCCATGCTGTTT

hLIN28	Forward	CGGGCATCTGTAAGTGGTTC
	Reverse	CAGACCCTTGGCTGACTTCT

hNANOG	Forward	CCTATGCCTGTGATTTGTGG
	Reverse	TTCTCTGCAGAAGTGGGTTG

hNFIC	Forward	CGACTTCCAGGAGAGCTTTG
	Reverse	GTTCAGGTCGTATGCCAGGT

hGAPDH	Forward	CCATGGAGAAGGCTGGGG
	Reverse	CAAAGTTCTCATGGATGACC

## Data Availability

The data that support the findings of this study are available from Dong-Seol Lee (iceburge@snu.ac.kr) upon reasonable request.

## Supplementary Materials

Supplementary 1 Supplementary Figure 1. The mandibles of WT and *Nfic*^−/−^ mice were examined using micro-CT, and the sagittal sections were stained with hematoxylin and eosin. (a) Representative micro-CT images of WT and (B) *Nfic*^−/−^ mandibles. The mutant mandible shows lower bone density than the WT. (c–e) Histological analyses of the WT and *Nfic*^−/−^ cervical loop using hematoxylin and eosin (H&E) staining. The cervical loop of the WT shows typical development on the labial side with an organized structure, whereas (f–h) the cervical loop of the *Nfic* mutant shows a disorganized structure. (d–e) and (g–h) are higher magnifications of boxed C and F, respectively. (a–h) 6 weeks. Scale bars = (c) and (f) = 500 *μ*m; (d) = 200 *μ*m; (g) = 100 *μ*m; (e) and (h) = 50 *μ*m. Abbreviations: DM: dental mesenchyme; IEE: inner enamel epithelium; LaCL: labial cervical loop; LiCL: lingual cervical loop; OEE: outer enamel epithelium; SR: stellate reticulum; TA: transit-amplifying cells; WT: wild type.

Supplementary 2 Supplementary Figure 2. Observations of fibroblast cells harvested from the tongues of WT and *Nfic*^−/−^ mice under a light microscope. Abbreviations: WT: wild type.

## References

[1] Gronthos S., Brahim J., Li W. (2002). Stem cell properties of human dental pulp stem cells. *Journal of Dental Research*.

[2] Smith C. E., Warshawsky H. (1975). Cellular renewal in the enamel organ and the odontoblast layer of the rat incisor as followed by radioautography using 3H-thymidine. *The Anatomical Record*.

[3] Gan L., Liu Y., Cui D. X., Pan Y., Wan M. (2020). New insight into dental epithelial stem cells: identification, regulation, and function in tooth homeostasis and repair. *World J Stem Cells*.

[4] Harada H., Kettunen P., Jung H. S., Mustonen T., Wang Y. A., Thesleff I. (1999). Localization of putative stem cells in dental epithelium and their association with notch and FGF signaling. *The Journal of Cell Biology*.

[5] Kuang-Hsien Hu J., Mushegyan V., Klein O. D. (2014). On the cutting edge of organ renewal: identification, regulation, and evolution of incisor stem cells. *Genesis*.

[6] Gronostajski R. M. (2000). Roles of the NFI/CTF gene family in transcription and development. *Gene*.

[7] Lee D. S., Choung H. W., Kim H. J. (2014). NFI-C regulates osteoblast differentiation via control of osterix expression. *Stem Cells*.

[8] Steele-Perkins G., Butz K. G., Lyons G. E. (2003). Essential role for NFI-C/CTF transcription-replication factor in tooth root development. *Molecular and Cellular Biology*.

[9] Lee D. S., Park J. T., Kim H. M. (2009). Nuclear factor I-C is essential for odontogenic cell proliferation and odontoblast differentiation during tooth root development∗. *The Journal of Biological Chemistry*.

[10] Lee D. S., Roh S. Y., Park J. C. (2018). The Nfic-osterix pathway regulates ameloblast differentiation and enamel formation. *Cell and Tissue Research*.

[11] Zhang J., Wang Z., Jiang Y. (2015). Nuclear factor I-C promotes proliferation and differentiation of apical papilla-derived human stem cells in vitro. *Experimental Cell Research*.

[12] Lee D. S., Roh S. Y., Choi H., Park J. C. (2020). NFI-C is required for epiphyseal chondrocyte proliferation during postnatal cartilage development. *Molecules and Cells*.

[13] Lefebvre V., Dumitriu B., Penzo-Méndez A., Han Y., Pallavi B. (2007). Control of cell fate and differentiation by Sry-related high-mobility-group box (sox) transcription factors. *The International Journal of Biochemistry & Cell Biology*.

[14] Arnold K., Sarkar A., Yram M. A. (2011). Sox2(+) adult stem and progenitor cells are important for tissue regeneration and survival of mice. *Cell Stem Cell*.

[15] Avilion A. A., Nicolis S. K., Pevny L. H., Perez L., Vivian N., Lovell-Badge R. (2003). Multipotent cell lineages in early mouse development depend on SOX2 function. *Genes & Development*.

[16] Juuri E., Jussila M., Seidel K. (2013). Sox2 marks epithelial competence to generate teeth in mammals and reptiles. *Development*.

[17] Juuri E., Saito K., Ahtiainen L. (2012). Sox2+ stem cells contribute to all epithelial lineages of the tooth via Sfrp5+ progenitors. *Developmental Cell*.

[18] Masui S., Nakatake Y., Toyooka Y. (2007). Pluripotency governed by Sox2 via regulation of Oct3/4 expression in mouse embryonic stem cells. *Nature Cell Biology*.

[19] Sanz-Navarro M., Seidel K., Sun Z. (2018). Plasticity within the niche ensures the maintenance of a Sox2+ stem cell population in the mouse incisor. *Development*.

[20] Sun Z., Yu W., Sanz Navarro M. (2016). Sox2 and Lef-1 interact with Pitx2 to regulate incisor development and stem cell renewal. *Development*.

[21] Basu-Roy U., Ambrosetti D., Favaro R., Nicolis S. K., Mansukhani A., Basilico C. (2010). The transcription factor Sox2 is required for osteoblast self-renewal. *Cell Death and Differentiation*.

[22] Yoon D. S., Kim Y. H., Jung H. S., Paik S., Lee J. W. (2011). Importance of Sox2 in maintenance of cell proliferation and multipotency of mesenchymal stem cells in low-density culture. *Cell Proliferation*.

[23] Liu P., Cai J., Dong D. (2015). Effects of SOX2 on proliferation, migration and adhesion of human dental pulp stem cells. *PLoS One*.

[24] Takahashi K., Yamanaka S. (2006). Induction of pluripotent stem cells from mouse embryonic and adult fibroblast cultures by defined factors. *Cell*.

[25] Thesleff I., Vaahtokari A., Partanen A. M. (1995). Regulation of organogenesis. Common molecular mechanisms regulating the development of teeth and other organs. *The International Journal of Developmental Biology*.

[26] Zhang Y. D., Chen Z., Song Y. Q., Liu C., Chen Y. P. (2005). Making a tooth: growth factors, transcription factors, and stem cells. *Cell Research*.

[27] Jernvall J., Thesleff I. (2000). Reiterative signaling and patterning during mammalian tooth morphogenesis. *Mechanisms of Development*.

[28] Amand T. R., Zhang Y., Semina E. V. (2000). Antagonistic signals between BMP4 and FGF8 define the expression of Pitx1 and Pitx2 in mouse tooth-forming anlage. *Developmental Biology*.

[29] Kettunen P., Laurikkala J., Itäranta P., Vainio S., Itoh N., Thesleff I. (2000). Associations of FGF-3 and FGF-10 with signaling networks regulating tooth morphogenesis. *Developmental Dynamics*.

[30] Harada H., Toyono T., Toyoshima K. (2002). FGF10 maintains stem cell compartment in developing mouse incisors. *Development*.

[31] Wang X. P., Suomalainen M., Felszeghy S. (2007). An integrated gene regulatory network controls stem cell proliferation in teeth. *PLoS Biology*.

[32] Yokohama-Tamaki T., Ohshima H., Fujiwara N. (2006). Cessation of Fgf10 signaling, resulting in a defective dental epithelial stem cell compartment, leads to the transition from crown to root formation. *Development*.

[33] Lee J. H., Lee D. S., Choung H. W. (2011). Odontogenic differentiation of human dental pulp stem cells induced by preameloblast-derived factors. *Biomaterials*.

[34] Yoo C. H., Na H. J., Lee D. S. (2013). Endothelial progenitor cells from human dental pulp-derived iPS cells as a therapeutic target for ischemic vascular diseases. *Biomaterials*.

[35] Seidel K., Ahn C. P., Lyons D. (2010). Hedgehog signaling regulates the generation of ameloblast progenitors in the continuously growing mouse incisor. *Development*.

[36] Smith C. E. (1980). Cell turnover in the odontogenic organ of the rat incisor as visualized by graphic reconstructions following a single injection of 3H-thymidine. *The American Journal of Anatomy*.

[37] Li J., Feng J., Liu Y. (2015). BMP-SHH signaling network controls epithelial stem cell fate via regulation of its niche in the developing tooth. *Developmental Cell*.

[38] Zhang X., Ibrahimi O. A., Olsen S. K., Umemori H., Mohammadi M., Ornitz D. M. (2006). Receptor specificity of the fibroblast growth factor family. The complete mammalian FGF family. *The Journal of Biological Chemistry*.

[39] Wang L., Su Y., Huang C. (2019). NANOG and LIN28 dramatically improve human cell reprogramming by modulating LIN41 and canonical WNT activities. *Biology open*.

[40] Humphreys B. D., Bonventre J. V. (2008). Mesenchymal stem cells in acute kidney injury. *Annual Review of Medicine*.

[41] Pisciotta A., Bertoni L., Vallarola A., Bertani G., Mecugni D., Carnevale G. (2020). Neural crest derived stem cells from dental pulp and tooth-associated stem cells for peripheral nerve regeneration. *Neural Regeneration Research*.

[42] Volponi A. A., Pang Y., Sharpe P. T. (2010). Stem cell-based biological tooth repair and regeneration. *Trends in Cell Biology*.

[43] Peng Z., Liu L., Wei X., Ling J. (2014). Expression of Oct-4, SOX-2, and MYC in dental papilla cells and dental follicle cells during in-vivo tooth development and in-vitro co-culture. *European Journal of Oral Sciences*.

[44] Zhang L., Yuan G., Liu H., Lin H., Wan C., Chen Z. (2012). Expression pattern of Sox2 during mouse tooth development. *Gene Expression Patterns*.

[45] Peng Z., Liu L., Zhang W., Wei X. (2021). Pluripotency of dental pulp cells and periodontal ligament cells was enhanced through cell-cell communication via STAT3/Oct-4/Sox2 signaling. *Stem Cells International*.

[46] Lima R. L., Holanda-Afonso R. C., Moura-Neto V., Bolognese A. M., DosSantos M. F., Souza M. M. (2017). Human dental follicle cells express embryonic, mesenchymal and neural stem cells markers. *Archives of Oral Biology*.

[47] Roh S. Y., Park J. C. (2017). The role of nuclear factor I-C in tooth and bone development. *Journal of the Korean Association of Oral and Maxillofacial Surgeons*.

[48] Shu J., Wu C., Wu Y. (2013). Induction of pluripotency in mouse somatic cells with lineage specifiers. *Cell*.

[49] Hammachi F., Morrison G. M., Sharov A. A. (2012). Transcriptional activation by Oct4 is sufficient for the maintenance and induction of pluripotency. *Cell Reports*.

[50] Tsai C. C., Su P. F., Huang Y. F., Yew T. L., Hung S. C. (2012). Oct4 and Nanog directly regulate Dnmt1 to maintain self-renewal and undifferentiated state in mesenchymal stem cells. *Molecular Cell*.

[51] Chou B. K., Cheng L. (2013). And then there were none: no need for pluripotency factors to induce reprogramming. *Cell Stem Cell*.

[52] Lee H. K., Lee D. S., Park S. J., Cho K. H., Bae H. S., Park J. C. (2014). Nuclear factor I-C (NFIC) regulates dentin sialophosphoprotein (DSPP) and E-cadherin via control of Kruppel-like factor 4 (KLF4) during dentinogenesis. *The Journal of Biological Chemistry*.

[53] Malaab M., Renaud L., Takamura N. (2022). Antifibrotic factor KLF4 is repressed by the miR-10/TFAP2A/TBX5 axis in dermal fibroblasts: insights from twins discordant for systemic sclerosis. *Annals of the rheumatic diseases*.

