# Supplementary figures and images for: Nuclear Factor I-C Regulates Stemness Genes and Proliferation of Stem Cells in Various Mineralized Tissue through Epithelial-Mesenchymal Interactions in Dental Epithelial Stem Cells
Source: Stem Cells Int. 2022 Sep 27;2022:1092184. doi: 10.1155/2022/1092184 (PMC9533135; doi:10.1155/2022/1092184)

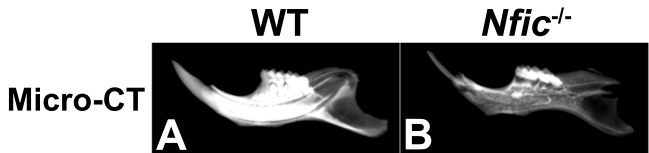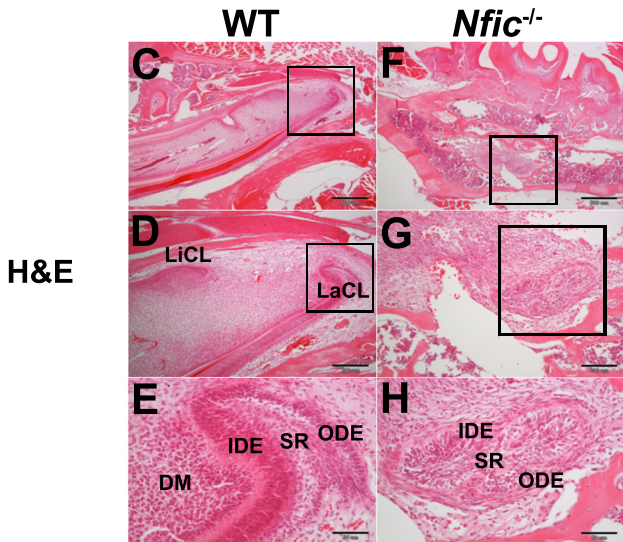

Supplement: Supplementary 1 — Supplementary Figure 1. The mandibles of WT and Nfic−/− mice were examined using micro-CT, and the sagittal sections were stained with hematoxylin and eosin. (a) Representative micro-CT images of WT and (B) Nfic−/− mandibles. The mutant mandible shows lower bone density than the WT. (c–e) Histological analyses of the WT and Nfic−/− cervical loop using hematoxylin and eosin (H&E) staining. The cervical loop of the WT shows typical development on the labial side with an organized structure, whereas (f–h) the cervical loop of the Nfic mutant shows a disorganized structure. (d–e) and (g–h) are higher magnifications of boxed C and F, respectively. (a–h) 6 weeks. Scale bars = (c) and (f) = 500 μm; (d) = 200 μm; (g) = 100 μm; (e) and (h) = 50 μm. Abbreviations: DM: dental mesenchyme; IEE: inner enamel epithelium; LaCL: labial cervical loop; LiCL: lingual cervical loop; OEE: outer enamel epithelium; SR: stellate reticulum; TA: transit-amplifying cells; WT: wild type. [file 1092184.f1.pdf]

**WT**

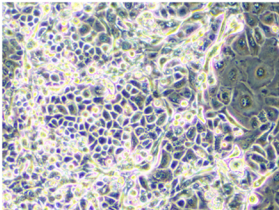

***Nfic*<sup>-/-</sup>**

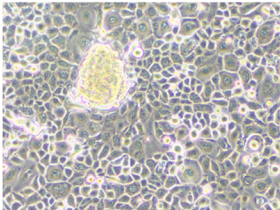

Supplement: Supplementary 2 — Supplementary Figure 2. Observations of fibroblast cells harvested from the tongues of WT and Nfic−/− mice under a light microscope. Abbreviations: WT: wild type. [file 1092184.f2.pdf]
